# A evidence-based approach to selecting post-exercise cryostimulation techniques for improving exercise performance and fatigue recovery: A systematic review and meta-analysis

**DOI:** 10.1016/j.heliyon.2024.e32196

**Published:** 2024-06-03

**Authors:** Chen Feng, Peng Chen, Wei Zhang, Bingting Luo, Geng Du, Ting Liao, Chanjuan Zheng

**Affiliations:** a Aquatic Therapy and Fitness Center, Wuhan Sports University, Wuhan, Hubei, China; b Hubei Provincial Hospital of Integrated Chinese & Western Medicine, Wuhan, Hubei, China; c School of Exercise and Health, Shanghai University of Sport, Shanghai, China

**Keywords:** Cryostimulation, Cold water immersion, Contrast water therapy, Whole-body cryostimulation

## Abstract

**Rationale:**

Cryostimulation involves using water environments and low temperatures as intervention mediums, with main methods including CWI (cold water immersion), CWT (contrast water therapy), and WBC (whole-body cryostimulation). Previous systematic reviews focused on the effect of cryostimulation on muscle fatigue and sports performance. However, studies on the selection of different cryostimulation methods and their intervention effects present inconsistent results.

**Introduction:**

To systematically review and methodologically appraise the quality and effectiveness of existing intervention studies that the effects of various cryostimulation methods, including CWI, CWT, and WBC, on exercise performance and fatigue recovery.

**Methods:**

Following PRISMA guidelines, we conducted searches in PubMed, Embase, The Cochrane Library, Web of Science, and EBSCO databases to gather randomized controlled trials or self-controlled trials involving CWI/CWT/WBC and their effects on exercise performance or fatigue recovery. The search period ranged from November 2013 to November 2, 2023. Literature screening was performed using EndNote X9.1, and the quality of included studies was assessed using the Cochrane risk of bias assessment tool. Meta-analysis was conducted using RevMan 5.3 software.

**Results:**

This study included a total of 18 articles, included a total of 499 healthy participants, comprising 479 males and 20 females. Among them, participants underwent cryostimulation, including 102 using CWT, using CWI, and 58 using WBC. Compared to the control group, cryostimulation can significantly alleviate muscle pain intensity (SMD -0.45, 95% CL -0.82 to 0.09, P = 0.01). Specifically, CWI significantly reduced muscle pain intensity (SMD = −0.45, 95% CI: 0.820.09, P = 0.01), WBC significantly decreased C-reactive protein levels (SMD = −1.36, 95% CI: 2.350.36, P = 0.008). While, CWT showed no significant differences from the control group in exercise performance and fatigue recovery indicators (P > 0.05).

**Conclusion:**

Cryostimulation can significantly reduce muscle pain intensity and perceived fatigue. Specifically, CWI significantly alleviates muscle pain intensity, WBC significantly lowers markers of inflammation caused by fatigue after exercise, in contrast, CWT does not significantly improve exercise performance and fatigue recovery. After exercise, compared with rest, using cryostimulation may have more noticeable benefits for muscle fatigue and muscle pain, with recommendations prioritizing WBC and CWI particularly for addressing inflammation and muscle pain. However, all cryostimulation may have no significant influence on exercise performance.

## 1 Introduction

Previous sports research has predominantly focused on identifying the most effective training methods to maintain and improve athletic performance [1]. However, maximizing athlete performance depends not only on adopting an efficient training technique but also on achieving an optimal balance between training and recovery [2] In real-world situations, elite athletes often undergo high-intensity interval training and intense training loads, with the brief intervals between competitive events usually being insufficient for complete physiological recovery [3,4]. Moreover, competition and training activities can induce eccentric contractions and tissue vibration, leading to muscle damage from structural protein breakdown in the muscle fibers and/or connective tissues [5,6]. Subsequently, tissue inflammation, delayed onset muscle soreness (DOMS), and an increased perception of fatigue may occur in the recovery phase post-exercise. DOMS is also closely associated with concentration changes [5,7]in observable serum markers, such as creatine kinase (CK) [5], as well as the inflammatory biomarkers C-reactive protein (CRP) [6]and interleukin-6 (IL-6) [8]. The changes in these exercise-induced inflammatory factors may temporarily result in decreased muscle strength, disrupted joint proprioception, diminished physical performance, or an increased injury risk [4,9,10]. In such circumstances, optimizing the recovery period is crucial for coaches and athletes to maintain peak athletic performance, particularly during critical time points such as the competitive season.

Cryostimulation therapy encompasses the application of water and low temperatures as an intervention medium, with the primary methods comprising cold-water immersion (CWI), contrast water therapy (CWT), and whole-body cryostimulation (WBC) [9]. In recent years, researchers have explored cryostimulation as a recovery technique to enhance athletic performance and muscle fatigue recovery. Among these methods, CWI acts via local vasoconstriction and hydrostatic pressure [11,12], while CWT utilizes the temperature difference and mechanical properties of water to achieve therapeutic effect [13,14]. In the case of WBC, this technique alters the tissue temperature and blood flow of the human body through extremely low temperatures, thereby regulating the inflammatory factor levels after exercise [15]. Although cryostimulation following exercise has become a widespread practice, research findings concerning the choice of different methods have been inconsistent [16]. Additionally, the physiological mechanisms by which distinct cryostimulation methods affect athletic performance warrant further elucidation, with their intervention effects also remaining unclear [3].

Therefore, this review systematically investigated the impact of the cryostimulation methods of CWI, CWT, and WBC on athletic performance and fatigue recovery, aiming to provide evidence-based guidance for exploring optimal recovery methods, promoting fatigue recovery, and augmenting athletic performance.

## 2 Methods

### 2.1 Information sources and search strategy

Search Strategy following PRISMA Guidelines [17]. We referred to databases searched for many high-quality meta-analyses [[18], [19], [20]], So we search Databases are PubMed, Embase, The Cochrane Library, Web of Science and EBSCO. Keywords: 1) cryostimulation OR cold water immersion OR contrast water therapy OR whole-body cryostimulation; 2) sport performance; 3) fatigue recovery. Search Period is November 2010 to November 2023. Latest Update is November 2, 2023.

Taking Pubmed as an example, our literature search formula is as follows:

((“Cryotherapy" [Mesh]) OR (Cryotherapies [Title/Abstract]) OR (Cold Therapy [Title/Abstract]) OR (Cold Therapies [Title/Abstract]) OR (Cold water immersion [Title/Abstract]) OR (Contrast water therapy [Title/Abstract]) OR (Whole-body cryotherapy [Title/Abstract])) AND ((“Myalgia" [Mesh]) OR (Muscle soreness [Title/Abstract]) OR (Muscle pain [Title/Abstract]) OR (Delayed onset muscle soreness [Title/Abstract]) OR (Perceived fatigue [Title/Abstract]) OR (Perceived recovery [Title/Abstract]) OR (Muscle strength [Title/Abstract]) OR (Power [Title/Abstract]) OR (Creatine kinase [Title/Abstract]) OR (Interleukin-6 [Title/Abstract]) OR (C-reactive protein [Title/Abstract]))

### 2.2 Selection variables

Peak power refers to the maximum power that the human body can achieve during exercise, and is an important predictive indicator of explosive force during exercise [21]. Horizontal hop distance/vertical hop height is considered an easy-to-implement and valuable indicator of muscle strength [22], a significant predictor of maximum speed and explosive force in sports, and a useful method for assessing mobility and functional capacity in athletes [23]. Therefore, Peak Power and Horizontal Hop Distance/Vertical Hop Height have been included in the meta-analysis to represent the impact on exercise performance following cryostimulation.

Creatine Kinase (CK) in the plasma is considered a byproduct of muscle fatigue after exercise. C-reactive protein (CRP)/Interleukin-6(IL-6) are markers of inflammation caused by fatigue after exercise. CK, CRP, IL-6 serve as objective indicators of fatigue changes. In addition, perceived recovery and perceived fatigue reflect fatigue recovery from an individual's subjective experience.

### 2.3 Eligibility criteria

**Fig. 1:**
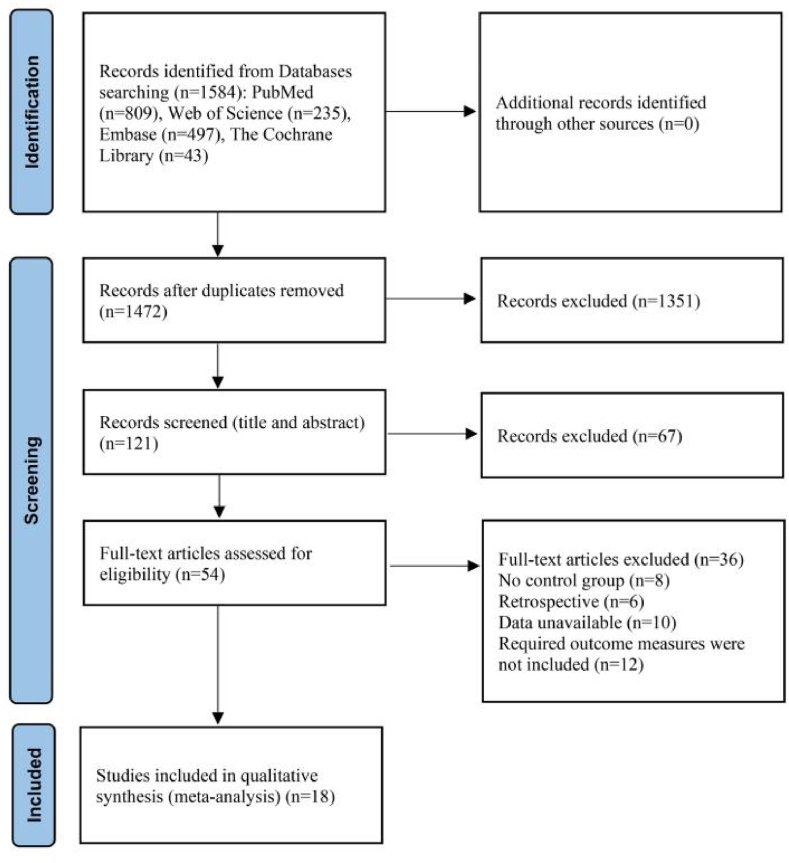
Flow-chart describing the systematic review procedure.

To be eligible for inclusion, studies needed to satisfy the following conditions: (1) implementation of an exercise intervention succeeded by a recovery intervention with comprehensive details on the procedures, encompassing the exercise modality, duration, and intensity; (2) explicit delineation of the modality and timing of the recovery intervention, excluding repeated sessions of recovery and/or combined modalities (e.g., ice + massage); (3) outcomes comprising valid tests and measures of Delayed Onset Muscle Soreness (DOMS), perceived fatigue, muscle damage, and inflammation markers, with the study population consisting of healthy adults aged >18 to < 65 years; and (4) reporting the number of participants and all essential data for effect size calculations. Studies were excluded if they reported results from a previously published source (duplicated data). Our study protocol adhered to the Quality of Reporting of Meta-analyses (QUOROM) guidelines [24] and the Preferred Reporting Items for Systematic Reviews and Meta-Analyses (PRISMA) statements [25]. The exclusion criteria are detailed in Fig. 1.

### 2.4 Data analysis

Statistical analysis was performed using RevMan 5.3 software, and conducted by two researchers (CF and PC), who independently screened literature, extracted data, and cross-verified the results. In case of disagreements, a third party was consulted to assist in reaching a consensus. For all outcome measures in this study, which were continuous data, if the outcomes were assessed on the same scale and in the same units, the effect size was calculated using the weighted mean difference (WMD) as the effect measure. If different scales and units were used, the standardized mean difference (SMD) was employed as the effect measure. All effect sizes were expressed with a 95% confidence interval (CI), and a significance level of P < 0.05 indicated a statistically significant difference [25]. Heterogeneity analysis was conducted for the included studies. When P > 0.1 and I^2^<50%, it indicated homogeneity among the studies, and a fixed-effects model was used for the meta-analysis. Conversely, if there was heterogeneity among the studies (P ≤ 0.1 or I^2^ ≥ 50%), a random-effects model was applied for the meta-analysis. Subgroup analysis or sensitivity analysis was performed to identify the sources of heterogeneity.

### 2.5 Risk of bias

Two researchers (CF and PC) assessed the risk of bias in the included studies using the risk of bias assessment tool for Randomized Controlled Trials (RCTs) as outlined in the Cochrane Handbook [16]. The evaluation criteria encompassed the following aspects: generation of random sequences; allocation concealment; implementation of blinding for participants and researchers; blinding assessment for study outcomes; completeness of outcome data; blinding of outcome assessors; selective reporting of study results; and other potential sources of bias. Each criterion was assigned one of three levels: ‘high risk’,‘low risk’, or ‘unclear’.

## 3 Results

### 3.1 Risk of bias of included studies

**Fig. 2:**
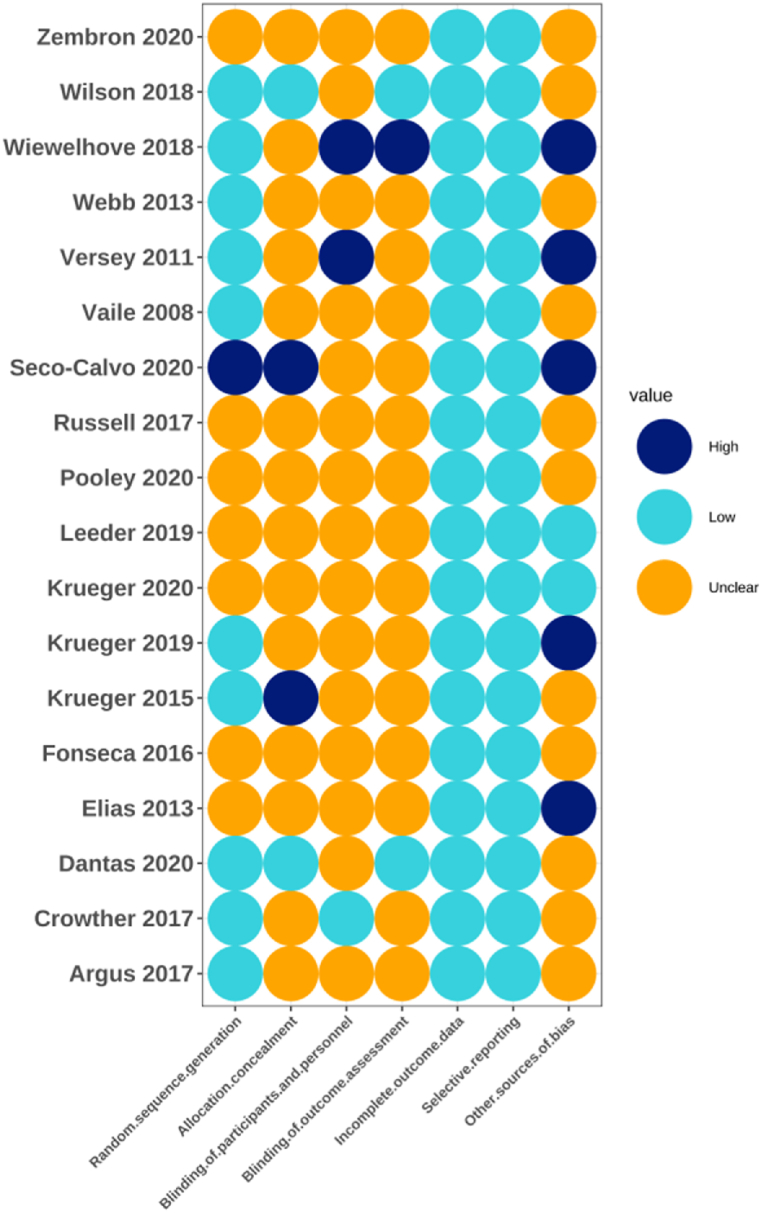
Risk of bias graph for all included studies.

**Fig. 3:**
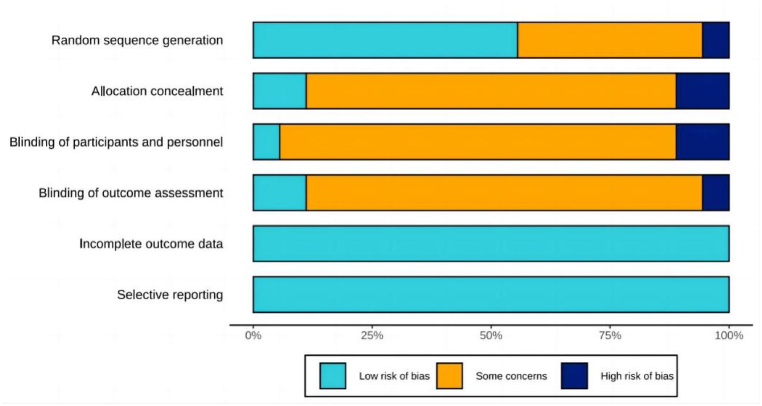
Risk of bias graph for all included studies.

Among the 18 included studies, the specific random sequence generation methods used were described in nine studies, while only the implementation of randomization was mentioned in the remaining nine. Furthermore, only one study employed allocation concealment, while three conducted single blinding. No dropouts were reported in all 18 studies. Lastly, the risk of bias for all included studies has been plotted in Fig. 2, Fig. 3.

### 3.2 Characteristics of included studies

This study included 18 studies that investigated the effects of varied cryostimulation methods on athletic performance and fatigue recovery. Six studies [12,14,26,27,27,28] reported peak power, while five [11,14,[27], [28], [29]] recorded jump height/distance. Further, eight studies [14,14,26,26,29,[27], [28], [29]] assessed muscle pain intensity, and two [11,29] determined perceived fatigue. Additionally, perceived recovery was measured in four studies [11,14,26,28], and CK levels in six [11,14,26,27,27,28]. Similarly, CRP levels were described in three studies [12,15,28], whereas three [12,27,28] determined IL-6 levels. In terms of sex distribution among the total 499 healthy participants from all studies, 479 were males and 20 were females. Of them, 269 participants underwent cryostimulation, wherein CWT, CWI, and WBC were administered in 102, 109, and 58, respectively. Complete details of the study characteristics are provided in Table 1.

**Table 1:** Summary of the included studies.

Author, Year	Sample size	Fatigue induction protocol	Intervention	Outcome indicators
Elias 2013 [30]	CWT: 8 (8 M) CWI: 8 (8 M) Con: 8 (8 M)	a full practice match with a total match duration of 75 min	CWT:12 °C/7min, 38 °C/7min CWI: 12 °C/14min Con: seated/14 min	muscle pain intensity, perceived fatigue
Versey 2011 [31]	CWT: 11 (11 M) Con: 11 (11 M)	a cycling protocol with 75 min	CWT:14.6 ± 0.3 °C/6min, 38.4 ± 0.6 °C/6min Con: seated	peak power
Crowther 2017 [32]	CWT: 34 (34 M) CWI: 34 (34 M) Con: 34 (34 M)	a 45 min simulated team-game circuit	CWI:15 °C/14min CWT:15 °C/7min,38 °C/7min Con: Outdoor jogging	muscle pain intensity, peak power, perceived recovery
Wilson 2017 [33]	CWI: 10 (10 M) WBC: 11 (11 M) Con: 10 (10 M)	marathon	CWI: 8 °C/10min WBC: 85 ± 5 °C/3min Con: taking a tart cherry juice supplement	muscle pain intensity
Argus 2016 [34]	CWI: 13 (13 M) CWT: 13 (13 M) Con: 13 (13 M)	a resistance training session with 55min	CWI: 15 °C/14min CWT:15 °C/1min,38 °C/1min Con: seated/14 min	muscle pain intensity, perceived fatigue
Krueger 2019 [35]	CWI: 9 (9 M) Con: 9 (9 M)	a friendly match with 60min	CWI: 5–8 °C/5min Con: seated	perceived recovery
Leeder 2019 [36]	CWI: 11 (11 M) Con: 10 (10 M)	Loughborough Intermittent Shuttle Test on 3 occasions	CWI: 14 °C/14min Con: seated/14min	C-reactive protein, interleukin-6
Pooley 2019 [37]	CWI: 15 (15 M) Con: 15 (15 M)	soccer game with 80min	CWI: 14 °C/10min Con: 10 min low intensity exercise	muscle pain intensity, creatine kinase, jump height
Webb 2013 [14]	CWI: 21 (21 M) CWT: 21 (21 M) Con: 21 (21 M)	professional rugby league matches	CWI:10–12 °C/5min CWT:8–10 °C/1min,40–42 °C/2min Con: low-intensity exercise/7min	muscle pain intensity, creatine kinase, jump height
Vaile 2007 [31]	CWI: 12 (12 M) CWT: 15 (15 M)	eccentric bilateral leg press contractions	CWI: 15 °C/14min CWT:15 °C/7min,38 °C/7min	peak power, muscle strength, creatine kinase, interleukin-6
Wiewelhove 2018 [38]	CWI: 11 (11 M) Con: 12 (12 M)	a half-marathon	CWI: 15 °C/15min Con: seated/15min	muscle pain intensity, jump height, creatine kinase, interleukin-6, C-reactive protein
Russell 2017 [26]	WBC: 14 (14 M) Con: 14 (14 M)	repeated sprint exercise	WBC: 60 °C/30s, −135 °C/2min Con: seated	peak power, muscle pain intensity, creatine kinase, perceived recovery
Dantas 2019 [29]	CWI: 10 Con: 10	10-km run	CWI: 10 °C/10min Con: rested on foot outside/10min	muscle pain intensity, jump distance
Zembron 2020 [15]	WBC: 11 (11 M) Con: 9 (9 M)	training camp	WBC: 120 °C/6min Con: seated/6min	C-reactive protein
Fonseca 2016 [11]	CWI: 4 (4 M) Con: 4 (4 M)	training protocol with 40min	CWI: 6 °C/16min	creatine kinase, jump height
Seco-Calvo 2020 [12]	CWI: 12 (4 M) Con: 16 (4 M)	a full season of competition	CWI: 10.5 °C/10min	peak power
Krueger 2019 [27]	WBC: 11 Con: 11	high-intensity running	WBC: 10 °C ∼ -110 °C/3min Con: active recovery	interleukin-6
Krueger 2015 [28]	WBC: 11 (11 M) Con: 11 (11 M)	high-intensity running	WBC: 10 °C ∼ -110 °C/3min Con: active recovery	perceived recovery

CWI = cold water immersion; CWT = contrast water therapy; WBC = whole-body cryostimulation; M: male; F: female.

### 3.3 Characteristics of exercise protocols

Fatigue induction protocols included match scenarios [7,11,14], cycling [27], marathon running [26,28], fitness training [11,12,12,14,31,26,29], soccer games [1], rugby matches [14], and running [[27], [28], [29]]. Table 1 lists the complete information on the utilized exercise protocols.

### 3.4 Characteristics of cryostimulation

CWI, which represented the predominant cryostimulation method, involved immersing the lower limbs of the participants in cold water with temperatures of 5°C-14 °C [7,11,11,12,12,14,14,14,31,[27], [28], [29]], except for one study that utilized a water temperature of −10 °C [29]. The immersion duration typically ranged from 5 min to 16 min.

In the case of CWT, the entire body of the participants was alternatingly immersed in cold and hot water to leverage the temperature difference for therapeutic effects. The cold water temperatures were 8°C-14.7 °C, while the hot water temperatures were from 37.8 °C to 42 °C [7,14,31,29,27].

Finally, the WBC interventions employed liquid nitrogen and cold air, with the participants briefly exposed to extremely cold air in a specially designed cryochamber. The air temperatures ranged from −10 °C to −110 °C, while exposure time was 2 min–6 min [7].

### 3.5 Characteristics of the passive control group

Typically, participants in the passive control groups were instructed to sit on a chair in a room with a normal temperature of 6°C-32 °C and a relative humidity of 37%–62%.

### 3.6 Characteristics of exercise performance recovery

#### 3.6.1 Peak power

**Fig. 4:**
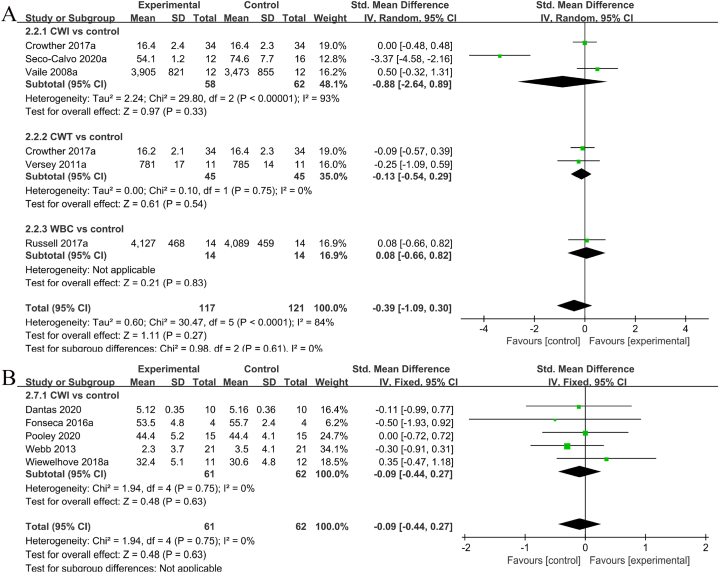
Forest plot comparing the exercise performance indicators between the cryostimulation and CON groups.

Five studies [12,14,31,26,27,28] reported the results of peak power. Substantial heterogeneity was observed among these studies (I^2^ = 84%, P < 0.001). Consequently, a random-effects model was employed for further analysis. After excluding one study [12], the heterogeneity significantly decreased (I^2^ = 0%, P = 0.75), while the results remained unchanged. Moreover, SMD was utilized for the combination owing to the varied measurement methods. The meta-analysis indicated that compared to the control group, the cryostimulation group did not exhibit a significant enhancement in peak power (SMD = −0.39, 95% CL: −1.09 to 0.30, 5 trails). In particular, no significant differences were found between the CWI and CON groups (SMD = −0.88, 95% CL: −2.64 to 0.89, 3 trails), CWT and CON groups (SMD = −0.13, 95% CL: −0.54 to 0.29, 2 trails), and WBC and CON groups (SMD = 0.08, 95% CL: −0.66 to 0.82, 1 trail). Part A of Fig. 4 depicts the details of peak power in the cryostimulation and CON groups.

#### 3.6.2 Horizontal jump distance/vertical jump height

Five studies [11,14,[27], [28], [29]] published the jump height/distance findings. Minimal heterogeneity was detected among the studies (I^2^ = 0%, P = 0.75); thus, a fixed-effects model was utilized for the analysis. Considering the distinct measurement methods, SMD was used for the combination. The meta-analysis revealed no significant difference between the CWI and CON groups (SMD = −0.09, 95% CL: −0.44 to 0.27, 4 trails). Details on jump height/distance in the cryostimulation and CON groups are available in part B of Fig. 4.

### 3.7 Objective characteristics of fatigue recovery

#### 3.7.1 CRP level

**Fig. 5:**
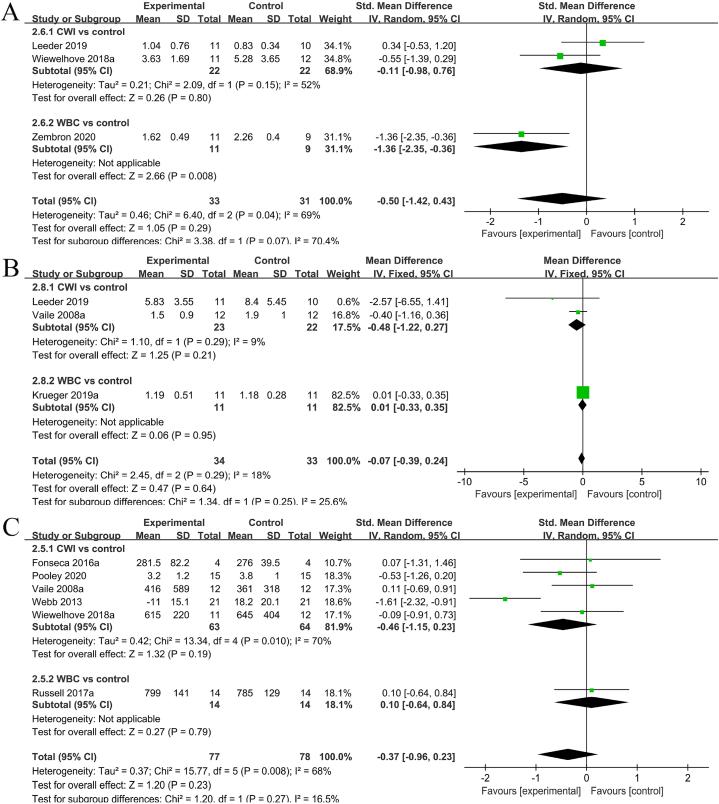
Forest plot comparing the objective characteristics of fatigue recovery between the cryostimulation and CON groups.

Three studies [12,15,28] described the CRP concentrations. Considerable heterogeneity was indicated among the studies (I^2^ = 76%, P = 0.02), resulting in a random-effects model analysis. Due to different measurement methods, SMD was employed for the combination. The meta-analysis results showed that the cryostimulation group had significantly reduced CRP levels compared to those in the control group (SMD = −0.50, 95% CL: −1.42 to 0.43, 3 trails). Subgroup analysis further demonstrated no significant difference between the CWI and CON groups (SMD = −0.11, 95% CL: −0.98 to 0.76, 2 trails), whereas a significant difference was found between the WBC and CON groups (SMD = −1.36, 95% CL: −2.35 to 0.36, 1 trail). Part A of Fig. 5 displays the information on CRP levels in the cryostimulation and CON groups.

#### 3.7.2 IL-6 level

Three studies [12,31,28] shared the IL-6 concentrations, with relatively low heterogeneity among them (I^2^ = 18%, P = 0.29). Consequently, a fixed-effects model analysis was performed. Furthermore, SMD was utilized for the combination due to consistent measurement methods. The meta-analysis suggested that compared to the control group, the cryostimulation group does not have significantly diminished IL-6 levels (SMD = −0.07, 95% CL: −0.39 to 0.24, 3 trails). Subgroup analysis indicated no significant differences between the CWI and CON groups (SMD = −0.48, 95% CL: −1.22 to 0.27, 2 trails) and the WBC and CON groups (SMD = −0.01, 95% CL: −0.33 to 0.35, 1 trail). See part B of Fig. 5 for details on IL-6 levels in the cryostimulation and CON groups.

#### 3.7.3 CK level

Six studies [11,14,31,26,27,28] mentioned the CK levels. Substantial heterogeneity was observed among the studies (I^2^ = 68%, P = 0.008), resulting in a random-effects model analysis. After excluding one study (Webb et al., in 2013), the heterogeneity significantly decreased (I^2^ = 75%, P = 0.10), while the results remained unchanged. Moreover, SMD was used for pooling because of the different measurement methods. The meta-analysis showed that compared to the control group, the cryostimulation group did not have significantly decreased serum CK levels (SMD = −0.37, 95% CI: −0.96 to 0.23, P = 0.23). Subsequent subgroup analysis suggested no significant difference between the CWI and CON groups (SMD = −0.46, 95% CL: −1.15 to 0.23, 5 trails) and the WBC and CON groups (SMD = 0.10, 95% CL: −0.64 to 0.84, 1 trail). Part C of Fig. 5 presents the details on CK levels in the cryostimulation and CON groups.

### 3.8 Subjective characteristics of fatigue recovery

#### 3.8.1 Muscle pain intensity rating

**Fig. 6:**
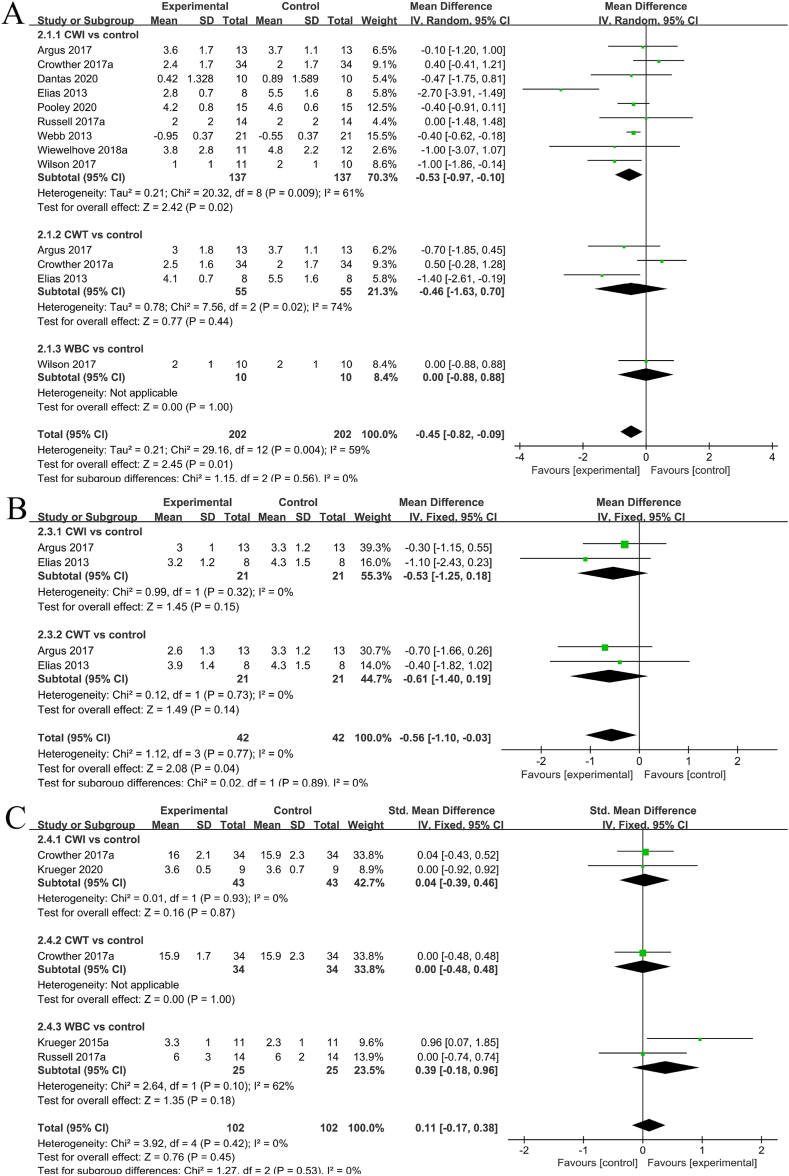
Forest plot comparing the subjective characteristics of fatigue recovery between the cryostimulation and CON groups.

Eight studies [14,14,26,26,29,[27], [28], [29]] provided the muscle pain intensity results. Notable heterogeneity was detected among the studies (I^2^ = 59%, P = 0.004), and a random-effects model analysis was conducted. After excluding the 2013 study by Elias, heterogeneity was significantly decreased (I^2^ = 26%, P = 0.19), with the results remaining unaltered. Based on the consistent measurement methods, SMD was employed for pooling. The meta-analysis demonstrated that compared to the control group, the cryostimulation group exhibited a significant alleviation of muscle pain intensity (SMD = −0.45, 95% CL: −0.82 to 0.09, 8 trails). Subgroup analysis revealed a significant difference between the CWI and CON groups (SMD = −0.53, 95% CL: −0.91 to −0.10, 8 trails). In contrast, no significant differences were observed between the CWT and CON groups (SMD = −0.46, 95% CL: −1.63 to 0.70, 3 trails) and the WBC and CON groups (SMD = −0.00, 95% CL: −0.88 to 0.88, 1 trail). See part A of Fig. 6 for information on the muscle pain intensity ratings in the cryostimulation and CON groups.

#### 3.8.2 Perceived fatigue rating

Two studies assessed perceived fatigue [11,29]. Minimal heterogeneity was detected between the studies (I^2^ = 0%, P = 0.77). Hence, a random-effects model analysis was performed. Additionally, SMD was used for pooling in light of the consistent measurement methods. The meta-analysis indicated that compared to the control group, the cryostimulation group experienced a significant improvement in perceived fatigue (SMD = −0.56, 95% CI: −1.1 to −0.03, 2 trails). Subgroup analysis further revealed significant differences between the CWI and CON groups (SMD = −0.53, 95% CL: −1.25 to 0.18, 2 trails) and the CWI and CON groups (SMD = −0.53, 95% CL: −1.25 to 0.18, 2 trails). The perceived fatigue ratings in the cryostimulation and CON groups are illustrated in part B of Fig. 6.

#### 3.8.3 Perceived recovery rating

Four studies [11,11,26,28] examined perceived recovery. Low heterogeneity was found between the studies (I^2^ = 0%, P = 0.42), resulting in a fixed-effects model analysis. Furthermore, owing to the distinct measurement methods, SMD was utilized for pooling. The meta-analysis indicated that compared to the control group, the cryostimulation group did not show significant improvement in perceived recovery (SMD = −0.11, 95% CL: −0.17 to 0.38, 4 trails). Subsequent subgroup analysis suggested significant differences between the CWI and CON groups (SMD = 0.04, 95% CL: −0.39 to 0.46, 2 trails), CWT and CON groups (SMD = 0.00, 95% CL: −0.48 to 0.48, 1 trail), and WBC and CON groups (SMD = 0.11, 95% CL: −0.17 to 0.38, 2 trails). Part C of Fig. 6 depicts the perceived recovery ratings in the cryostimulation and CON groups.

## 4 Discussion

This review to systematically review and methodological appraise the CWI, CWT, and WBC, on exercise performance and fatigue recovery. The results indicate cryostimulation can significantly reduce muscle pain intensity and perceived fatigue. In particular, the CWI technique leads to a significant alleviation of muscle pain intensity, while WBC can significantly lower the inflammation marker levels elevated by post-exercise fatigue. In contrast, CWT does not significantly improve exercise performance and fatigue recovery. Complete details of the summary of results are provided in Table 2.

**Table 2:** Summary of results.

	cryostimulation	CWI	CWT	WBC
Peak power	–	–	–	–
Horizontal hop distance/Vertical hop height	–	–	/	/
CRP	–	–	/	↓
IL-6	–	–	/	–
CK	–	–	/	–
Muscle pain	↓	↓	–	–
Perceived fatigue	↓	–	–	–
Perceived recovery	–	–	–	–

CWI: cold water immersion; CWT: contrast water therapy; WBC: whole-body cryostimulation; ↓ = significantly decrease in cryostimulation/CWI/CWT/WBC, groups, compared with CON, groups; ↑ = significantly increase in cryostimulation/CWI/CWT/WBC, groups, compared with CON, groups; - = unsignificant difference between cryostimulation/CWI/CWT/WBC, and CON, groups./= the meta-analysis results do not mention.

### 4.1 Methodological analysis of included literature

The risk of bias in most studies was either unclear or high, with this risk primarily arising from allocation concealment and experiment blinding. Only one study mentioned that participants were anonymized during grouping to conceal their treatment information from researchers [29]. This minimal allocation concealment may be due to the potential difficulty for researchers in censoring participant information during the administration of cryostimulation. Furthermore, only two studies confirmed that personnel were blinded to the participants [32,33], while one study indicated that the investigators were unaware of the participants' identities during outcome evaluation [29]. The limited blinding of the researchers may be attributed to the unavoidable direct contact between them and participants during the cryostimulation procedure. Therefore, future studies aiming to enhance methodological quality would benefit by incorporating more randomized controlled trials, allocation concealment, and blinded assessors in the research design.

### 4.2 Characteristics of exercise performance recovery

Power refers to the amount of work an individual can accomplish in a specific unit of time, and it serves as a physical quantity that represents the speed of performing work. Moreover, peak power in the context of physical activity is the maximum power that the human body can achieve during movement, acting as an important predictive indicator of muscle strength and explosive force [21]. Our study found that peak power did not significantly differ among the participants in the three cryostimulation groups and the control group.

Previous research showed that administering CWI following resistance training three times a week for 7 weeks did not elicit significant improvement in 1 R M bench press, 1 R M leg press, and peak push-up force [39]. Additionally, two other studies revealed no significant improvement in maximal isometric muscle strength after CWI intervention [33,34]. Similarly, CWT did not significantly augment maximal isometric muscle strength [34,40]. In contrast, research has suggested that WBC may have a positive impact on maximal muscle strength with a delayed effect, as evidenced by a decreased single-leg maximal eccentric contraction strength in the left knee at 24 and 48 h post-cryotherapy, followed by an increase in this parameter after 96 h [41]. In another study involving runners, immediate, 24-h, and 48-h post-exercise muscle strength displayed positive effects after WBC [42]. WBC may induce these improvements in muscle strength by alleviating pain and promoting fatigue recovery, two key factors in augmenting athletic performance. However, considering that cryostimulation administration immediately after exercise may introduce confounding effects of reduced muscle temperature on strength recovery [39], future research should directly compare the impact of different timings of cryostimulation post-exercise.

Jump height is a measure that reflects the recovery of lower limb explosive force and neuromuscular function. Several studies evaluating the effects of cryostimulation on jump height have presented conflicting results. Numerous studies have indicated that CWI intervention does not significantly improve jump height [35,43,44], whereas three studies have highlighted that CWT can significantly increase jump height [14,31,44]. Conversely, WBC did not exhibit a positive effect on post-exercise jump height and was even suggested to lower this measure below baseline levels [26,45].

Muscular explosive force is also a crucial aspect of athletic performance. The rate of force development (RFD), a crucial indicator of muscular explosive force related to neural activation, is calculated as the average slope of the force or torque-time curve during maximum effort. CWI has been reported to significantly improve RFD in squat jumps [33,46]. In contrast, RFD when jumping down from a 30-cm high step was shown to be lower in participants in the WBC group than those in the control group [24]. Sprinting speed is another critical performance measure of muscular explosive force, with studies indicating varied outcomes. Two studies demonstrated that CWI and CWT [32,44] do not significantly improve immediate short-distance sprinting speed. However, two other studies revealed that sprinting performance after CWI was superior to that in the control group within 24 h post-intervention [36,43]. Another study suggested that repeated sprint performance subsequent to CWT was superior to that in the control group within 48 h following therapy [30]. All these findings imply that CWI and CWT may exert positive effects on the speed and performance of muscular explosive force under different conditions and timeframes.

Some researchers have also elucidated the mechanisms of CWI therapy on muscle function. One such study showed that endurance training followed by CWI led to a decrease in the mTORC1 signaling pathway that regulated cell growth, while the level of the protein degradation marker FOX-O1 was elevated [39]. In another study of 4 weeks of high-intensity interval training and subsequent CWI on muscle cells, no change was detected in the expression levels of the molecules related to exercise performance; however, the markers of cellular oxidative stress response and mitochondrial biogenesis-associated signaling molecules were upregulated, while muscle cell oxidation was increased [47].

Based on these results, CWT may have a positive impact on the recovery of the muscular explosive force of the lower limbs, with the positive effects of CWI and CWT on speed recovery having a certain delayed effect. Finally, WBC may not be a suitable cryostimulation method for improving athletic performance recovery.

### 4.3 Objective characteristics of fatigue recovery

Muscle fatigue induces inflammatory marker production proportional to the degree of exercise-induced fatigue, enabling the objective assessment of fatigue recovery. Serum CK is a sensitive indicator of exercise intensity and can be used to estimate exercise-induced muscle cell damage. CK levels have been found to decrease after cryotherapy with CWI [36,37,29], CWT [[13], [14], [15]], and WBC [15]. Intense exercise can cause muscle tissue damage, manifesting as a rapid increase in CRP levels. Prior studies have shown that CRP levels after CWI [33] and WBC [15,33] interventions were significantly lower than those in the control group. TNF-α and IL-1β are immune cell products and indicators of cell-mediated immune activation, thereby indirectly reflecting the immune status of the body. Earlier investigations have reported that TNF-α and IL-1β levels after CWI intervention were lower than those in the control group [46,48]. In a study involving the application of CWI throughout the basketball season, myoglobin levels were significantly lower in the CWI group than in the control group [12]. The potential explanation for this effect is that the low temperature in CWI induces local vasoconstriction, thus attenuating the inflammatory response to muscle-damaging exercise and promoting recovery.

Further exploration of the effects of different cryostimulation methods has revealed specific differences. Lactic dehydrogenase (LDH) is a critical enzyme in anaerobic metabolism, wherein muscle cell damage and increased cell membrane permeability following intense exercise can result in heightened LDH concentrations. Two studies indicate that administering CWI after the competitions and training sessions of the athletes significantly diminishes LDH levels [11,12]. Conversely, compared to the control group, the CWT group showed a significant increase in LDH level and total white blood cell count 1 h after the intervention [40]. Moreover, IL-6, a known mediator of immune responses, is usually released immediately after exercise initiation and reaches its peak immediately after exercise. One study demonstrated that IL-6 levels following CWI were significantly lower than those in the control group [48], whereas other researchers revealed that IL-6 levels post-CWT did not significantly differ from those in the control group [31,49]. Testosterone, cortisol, and adrenaline are prominent hormones associated with exercise, with exercises of a certain intensity potentially elevating their levels. Previous research has suggested that cortisol and testosterone levels are lowered following CWI therapy [48,50]. Conversely, WBC for athlete recovery after soccer matches was shown to significantly elevate testosterone levels compared to those in the control group, along with no significant difference in cortisol levels [26]. Lactate is a metabolic product generated after high-intensity exercise, and a higher lactate clearance rate can lead to better cellular homeostasis maintenance and contribute to cellular function recovery. One study has reported that lactate levels following CWI intervention are significantly lower than those in the control group [51], whereas several others have indicated that CWI [43,48], CWT [44], and WBC [27] have no significant effect on lactate levels post-exercise. Furthermore, the accumulation and clearance of lactate after exercise are influenced by factors such as testing time, exercise mode, and individual health status [52], implying that comparisons between different studies may be affected by the diet, exercise, and the health of the participants.

In terms of the effects of cryostimulation mechanisms on the objective indicators of fatigue recovery, WBC significantly reduced IL-1β, hydrogen peroxide (H_2_O_2_), and nitric oxide (NO) levels [15]. Moreover, H_2_O_2_ and NO have contradictory roles in tissue repair processes. For example, these two molecules bind to growth factors and participate in muscle regeneration and repair [53,54]. In contrast, infiltrating neutrophils maintain high concentrations of H_2_O_2_ and NO locally, which can lead to further damage via oxidative injury, disrupt key structures in the excitation-contraction coupling process, and consequently delay complete recovery from injury [55].

Therefore, cryostimulation methods (i.e., CWI, CWT, and WBC) can be implemented as recovery measures after exercise to promote the clearance of certain fatigue-related biochemical markers. Among these methods, CWI can stimulate the clearance of various inflammatory markers, lactate, and hormones, thus achieving optimal recovery effects.

### 4.4 Subjective characteristics of fatigue recovery

The visual analog scale (VAS) is a commonly used self-assessment tool for measuring muscle soreness. Multiple studies have found that CWI [37,40], CWT [31], and WBC [38] can effectively reduce VAS scores by alleviating post-exercise muscle soreness. The Borg Rating of Perceived Exertion (Borg RPE) scale is a frequently administered self-assessment tool for evaluating fatigue. A few researchers have suggested that CWI and CWT [31,49] can significantly reduce the Borg RPE scale scores by effectively relieving post-exercise fatigue. Another study investigating the dose-response relationship of CWT for fatigue recovery after high-intensity cycling revealed that muscle fatigue and soreness improved after 6 min and 12 min of CWT compared to those in the control group, with CWT duration of below 12 min showing more significant effects than those lasting for ≥18 min [56]. Additionally, compared to the passive recovery group, the CWI and CWT groups demonstrated an overall reduction in perceived fatigue [57]. Cryostimulation timing is also a noteworthy factor, given that the application of CWI after exercise has been shown to reduce muscle soreness and fatigue at night, thereby improving sleep quality [43]. Moreover, one study reported that CWT 1 h after exercise-induced fatigue resulted in a more significant enhancement of perceptual recovery than CWI [32].

The effects of cryostimulation mechanisms on perceived fatigue recovery have also been examined in previous studies. For instance, CWI was determined to induce a series of physiological changes, such as decreased metabolic activity [54], reduced hormone secretion [48], and decreased blood flow in the limbs [58], via local vasoconstriction and alterations in hydrostatic pressure. Furthermore, the acute analgesic effect of CWI may be related to reduced neural conduction. In this mechanism, cold stimulation activates transient receptor potential melastatin 8 to subsequently inhibit signal input from pain receptors via spinal inhibitory interneurons or the direct inhibition of pain receptor signaling, ultimately modulating acute muscle soreness [59]. The therapeutic mechanism of CWT involves a pumping action generated by the changes in temperature-induced vasoconstriction and dilation, leading to reduced post-exercise swelling and aiding in lactate metabolism [60]. During CWT, participants may experience a stronger sense of comfort during the alternating immersion in hot water, potentially leading to a placebo effect [32]. Finally, the mechanism of WBC can be explained by the exposure of the body to cold stimulation that results in the production of β-endorphins (neurotransmitters with analgesic effects) and norepinephrine, thus relieving discomfort and providing analgesic effects [61].

All these findings collectively indicate that CWI, CWT, and WBC play a role in ameliorating perceived fatigue post-exercise. Although varied fatigue protocols, intervention frequencies, and individual factors, including physical fitness and psychological aspects, can influence experimental results and substantial bias may exist in subjective assessments, the existing research findings imply a positive effect of cryostimulation on perceived fatigue.

## 5 Limitations

Our study has several limitations that should be considered. 1) The inclusion of randomized controlled trials and self-controlled trials may have introduced heterogeneity in the results. 2) The relatively lower quality of a few included studies may have contributed to a certain risk of bias. 3) A few outcome measures were investigated in only a limited number of studies, potentially reducing the reliability of the evidence. 4) Our study did not fully consider the impact of muscle fatigue induction methods and the intervention period of cryostimulation. 5) A potential bias may have arisen due to the use of self-reported measures of muscle pain and fatigue, highlighting the need for more objective physiological markers in future research. 6) Our current study primarily focused on immediate and short-term outcomes, suggesting that future studies exploring the long-term effects of cryostimulation on athletic performance and recovery are required. 7) Lastly, the role of psychological factors, such as the placebo effect, should be considered when assessing the perceived efficacy of cryostimulation treatments, and the possible methods for controlling or accounting for these factors should be incorporated into the study design. Overall, acknowledging these shortcomings and factoring them when interpreting the results are crucial. Future research could address these limitations by enhancing the robustness and generalizability of the findings.

## 6 Conclusion

Cryostimulation can significantly reduce muscle pain intensity and perceived fatigue. In particular, the CWI technique leads to a significant alleviation of muscle pain intensity, while WBC can significantly lower the inflammation marker levels elevated by post-exercise fatigue. In contrast, CWT does not significantly improve exercise performance and fatigue recovery. Furthermore, compared to rest, cryostimulation after exercise may have more apparent benefits for muscle fatigue and muscle pain, with prior research recommending prioritizing WBC and CWI for specifically addressing inflammation and muscle pain.

Future research should include large-sample randomized controlled trials to enhance methodological quality. Additionally, comparing individual differences and cryostimulation technical parameters in experimental design is essential to determine the optimal cryostimulation method and application protocol.

## Funding

Staff management, computer use and publishing fee of this study were supported by the Humanities and Social Science Research Project of the Ministry of Education (No. 1926), Hubei Education Science and Technology Research Key Project (No. D20204103), Excellent Young and Middle-aged Scientific and Technological Innovation Teams in Hubei Universities (No. T202203), Hubei Science and Technology Innovation Special Project (No. 2021CFB359).

## Data availability statement

The data associated with our study has been deposited into a publicly available repository on Science Data Bank. https://cstr.cn/31253.11.sciencedb.16057.CSTR:31253.11.sciencedb.16057.

## CRediT authorship contribution statement

**Chen Feng:** Writing – review & editing, Writing – original draft, Project administration, Methodology, Investigation, Formal analysis, Data curation, Conceptualization. **Peng Chen:** Writing – original draft, Investigation, Formal analysis, Data curation, Conceptualization. **Wei Zhang:** Formal analysis, Data curation. **Bingting Luo:** Formal analysis, Data curation. **Geng Du:** Formal analysis, Data curation. **Ting Liao:** Writing – review & editing, Writing – original draft, Formal analysis, Data curation, Conceptualization. **Chanjuan Zheng:** Writing – review & editing, Writing – original draft, Formal analysis, Data curation, Conceptualization.

## Declaration of competing interest

The authors declare the following financial interests/personal relationships which may be considered as potential competing interests: Ting Liao reports financial support was provided by the Humanities and Social Science Research Project of the Ministry of Education (No. 1926), Hubei Education Science and Technology Research Key Project (No. D20204103). Chanjuan Zheng reports financial support was provided by Hubei Science and Technology Innovation Special Project (No. 2021CFB359). If there are other authors, they declare that they have no known competing financial interests or personal relationships that could have appeared to influence the work reported in this paper.
